# Chicago Society of Physicians and Surgeons

**Published:** 1873-11-15

**Authors:** 


Chicago Society of Physicians and Surgeons.—The regular meeting of the Society was held on Monday evening, Nov. 10, in a parlor of the Grand Pacific Hotel. There was an unusually large attendance of members present to listen to the extended and elaborate paper prepared by Dr. John Bartlett, on the Ague-plant, giving the results of his recent investigations.

The fungoid growth described by the Doctor was, he thought, the same plant discovered and described some years ago by Dr. Salisbury, who apparently however observed it only in its primary stages. Dr. Bartlett had traced some further and apparently previously undiscovered changes which take place, marking either a new stage of development of the same plant, or else the generation of a new and independent growth.

The Doctor exhibited three large colored plates to illustrate the different appearances presented by the growth, and also exhibited a number of the plants themselves under the microscope. He feels confident that they can be proved to occupy a causative relation to ague.

